# Quantitative Evaluation of Iranian Radiology Papers and Its Comparison with Selected Countries

**DOI:** 10.5812/iranjradiol.5050

**Published:** 2014-01-30

**Authors:** Mahyar Ghafoori, Hasan Emami, Abdolrasoul Sedaghat, Mohammad Ghiasi, Madjid Shakiba, Manijeh Alavi

**Affiliations:** 1 Department of Radiology, Hazrat Rasoul Akram Hospital, Iran University of Medical Sciences, Tehran, Iran; 2 Advanced Diagnostic and Interventional Radiology Research Center (ADIR), Tehran University of Medical Sciences, Tehran, Iran; 3 IT and Statistics Center, Shahid Beheshti University of Medical Sciences, Tehran, Iran; 4 Iranian Society of Radiology, Tehran, Iran; 5 Health Services Administration, Shahid Beheshti University of Medical Sciences, Tehran, Iran; 6 Deputy of Research and Technology, Ministry of Health and Medical Education, Tehran, Iran

**Keywords:** Societies, Scientific, Radiology, Geographic Locations

## Abstract

**Background:**

Recent technological developments in medicine, including modern radiology have promoted the impact of scientific researches on social life. The scientific outputs such as article and patents are products that show the scientists’ attempt to access these achievements.

**Objectives:**

In the current study, we evaluate the current situation of Iranian scientists in the field of radiology and compare it with the selected countries in terms of scientific papers. For this purpose, we used scientometric tools to quantitatively assess the scientific papers in the field of radiology.

**Materials and Methods:**

Radiology papers were evaluated in the context of medical field audit using retrospective model. We used the related databases of biomedical sciences for extraction of articles related to radiology. In the next step, the situation of radiology scientific products of the country were determined with respect to the under study regional countries.

**Results:**

Results of the current study showed a ratio of 0.19% for Iranian papers in PubMed database published in 2009. In addition, in 2009, Iranian papers constituted 0.29% of the Scopus scientific database. The proportion of Iranian papers in the understudy region was 7.6%.

**Conclusion:**

To diminish the gap between Iranian scientific radiology papers and other competitor countries in the region and achievement of document 2025 goals, multifold effort of the society of radiology is necessary.

## 1. Background

Nowadays, we are living in the postindustrial age and the age of science and communication. In this age, science and technology are important for survival and higher international improvement compared to all other assets. This is why we can see the worldwide extraordinary daily improvement in science and technology and the attention towards innovation. Fortunately, in the Islamic Republic of Iran, science, technology and innovation have been considered as the focus of attention. Recently, to achieve scientific and technologic development, various short term and long term plans have been designed and performed in Iran. The main goals of these plans are excellent and lead to a bright future for the people, and a better international position for the country. For example, we can mention preparing the Iranian 2025 vision document, the comprehensive scientific map of the country, and the scientific health map. In addition to these national programs, for the improvement of science and technology in Iran, assessment of these items in the level of scientific fields and disciplines is necessary for micro plans. This evaluation helps the scientists and government in programming technology development and leads to a more feasible macro plan.

Therefore, assessment of different scientific fields has been recommended for using the capacity and capabilities of experts, societies, and organizations. The first session of this program has been performed in 2008 with 30 medical scientific societies and for the second session, 40 scientific societies have been nominated by the Iranian deputy president in science and technology affairs for cooperation in this program. One of these societies is the society of radiology.

## 2. Objectives

The goal of the current study was to evaluate the Iranian radiology papers in Pubmed and Scopus databases and its comparison with the United States of America and regional countries including central Asia, Caucasus, Middle East and adjacent countries including Azerbaijan, Afghanistan, Jordan, Armenia, Uzbekistan, United Arab Emirates, Bahrain, Pakistan, Tajikistan, Turkmenistan, Turkey, Syria, Palestine, Kyrgyzstan, Kazakhstan, Qatar, Kuwait, Georgia, Iraq, Oman, Saudi Arabia, Lebanon, Egypt, and Yemen.

## 3. Materials and Methods

The current study is a descriptive analytical study in which we used the retrospective model. To search radiology papers in PubMed, the appropriate and standard search strategy was used. For this reason, the related keywords of medical subject heading (MeSH) terms indicating the subjects and papers of the field were chosen and all were searched in the website of: http://www.ncbi.nlm.nih.gov/pubmed. The option of MeSH database was selected in this website. The word “radiology” was entered in the search box and using the tab of “send to” and selecting the “Search box with OR”, it was added to the search strategy. All papers in the time interval of 2009 were searched. Then the “AND Iran [affiliation]” was added to the above mentioned strategy and the search was done again. This process was repeated for all under study region countries and for the USA as the pioneer of Radiology in the world. These processes were repeated exactly in the Scopus database.

## 4. Results

According to the results of the present study, in 2009, the proportion of Iranian articles in the field of radiology to all articles was 0.19% (source of PubMed). The proportion of articles in the field of radiology to all Turkish and USA articles were 0.85% and 35%, respectively. Iranian papers comprised 7.6% of the regional papers, while this figure was 34.6% for Turkey. Iranian papers made up 0.29% of the papers of the Scopus database in 2009, while this figure was 0.38% for Turkish articles and 1.49% for the USA. Iranian papers comprised up to 15.99% of the under study region papers and this percentage was 20.76% for Turkey (Table 1 and 2).

**Table 1.** Total Number of Papers Related to the Field of Radiology Published in PubMed in 2009

Row	Subject	Number
**1**	all (radiology) and limit-to (year “2009”)	1049
**2**	Iran [affiliation]	2
**3**	Turkey [affiliation]	9
**4**	Regional	26
**5**	USA [affiliation]	373

**Table 2.** Total Number of Papers Related to the Field of Radiology Published in Scopus in 2009

Row	Subject	Number
**1**	ALL(radiology)and limit-to (year “2009”)	82520
**2**	Iran [affiliation]	244
**3**	Turkey [affiliation]	317
**4**	Regional	1526
**5**	USA [affiliation]	1231

## 5. Discussion

Continual evaluation and improvement of scientific research is necessary in the field of radiology, because radiology has a unique position in the efficiency of diagnosis and treatment. In the present study, we examined the position of scientific outputs of the field of radiology among other biomedical disciplines in Iran and some selected countries. We used formal methodologies of records retrieval from database based on the scientometric protocols (1). According to the results of the current study, Iranian researches in radiology discipline are mainly in the clinical field and there are few studies on management assessment. The results showed Iranian products comprise 2% of the papers in PubMed scientific database in 2009, while this figure is more than fourfold for Turkey as our main regional competitor. Compared to twenty other countries in the region, our country constitutes 7.7% of the scientific production of the region. Moreover, paper production in comparison to the United States is 0.53%. Regarding paper production in the world, our country’s share in the Scopus scientific database is 0.29%. This figure in comparison to PubMed database shows a growth of 0.9% while the United States shows a considerable decline from 35% to 1.49%. Turkey consists 0.38% of this database showing a decrease greater than twice in comparison to PubMed scientific database (0.88%). The contribution of twenty regional countries in Scopus scientific database is 1.8% and Iran comprises 15.9% of the regional papers which is more than twice than PubMed scientific database.

In the next step of this study, we evaluated all aspects of radiology and its performance in Iran. These evaluations were divided into three groups including structure evaluation, process evaluation and outcome evaluation. Structure evaluation refers to what we do practically such as management structure, equipment, human resources, and education. Process evaluation includes the process of quality management, waiting time, and protocol (2, 3). Outcome evaluation includes aspects such as medical consequences and patient satisfaction (4, 5).

Roubidoux et al. (6) state that an evaluation is meaningless without standards. Evaluation is not the sampling process and unlike a study that has been designed for yielding statistics, is carried out for service quality improvement (7). As health care providing organizations are responsible for their continuous service quality improvement, a professional and appropriate clinical evaluation is an effective tool for patient care improvement and its results (8). Clinical evaluation in radiology and other medical professions is a regulatory need (9). Thus, for health care improvement, professional and organizational responsibility is considered in performing clinical evaluation in all aspects. According to Scherer et al., audit is defined as “regular and systematic testing or review of radiologic methods that seeks service quality improvement and patient health care” (10). The results of the current study showed evaluation has a significant role in service quality improvement; therefore, it should be considered very important. As Kyes et al. (11) mentioned the fact that radiology centers should consider evaluation as a strategic opportunity not a punishment; accordingly, they should continuously cooperate with the appraisers. They also stated that the evaluation should not be limited to one session, but performed continuously (11).

According to the above mentioned data in the result section, it is obvious that our scientific products are meaningfully lower in comparison to the under study region especially turkey as the main competitor. On the other hand, achievement of the 2025 prospect that is reaching the first position in the under study region needs double fold efforts now. Considering the fact that we have expert professors of radiology in Iran, this effort is achievable.

To reach the prospect horizon in the remaining time, our country should at least reach a four-fold increase in its scientific products compared to the current situation considering the regional country efforts and it should also accomplish a great endeavor. In this regard and for achievement of this goal, the society of radiology should adopt an active strategy. The society of radiology should prepare facilities to gather the researchers’ scientific activity data, develop journals in the field of radiology and index them in the valid international databases, increase the proportion of papers in the PubMed database via consultation with interface institutions, facilitating the publishing process in valid international databases, considering corporeal and spiritual incentives for researchers in order to encourage them in research and preparing the required conditions for those who do not have enough time for presenting their achievements as papers.

In examining the data presented here, it is important to note that the feedback provided by representatives of a small number of journals suggested an initial unwillingness to provide this data for endpoint publication use (9). Confidentiality issues and the premise that such information may be unjustly framed, potentially reflecting poorly on the standing of individual journals, were given as expressed concerns (10). For this reason, radiology evaluation in under study region showed main researches are in the clinical field and there is a few works on the management evaluation. In addition, evaluation employs a special method in which performance is compared with predefined standards (1).

In conclusion, it is obvious that promotion of radiology in Iran similar to developed countries and multilateral development is definitely needed. Therefore, development of scientific tendencies of the field could be considered as the society’s future goals that need appropriate processes. This goal is achieved by developing connections with international scientific societies.
